# Supplementary material for: Identification of photoperiod-induced specific miRNAs in the adrenal glands of Sunite sheep (Ovis aries)
Source: Front Vet Sci. 2022 Jul 22;9:888207. doi: 10.3389/fvets.2022.888207 (PMC9354845; doi:10.3389/fvets.2022.888207)
Supplement: Supplementary file 1 [file Data_Sheet_1.ZIP › Supplementary file/miRDeep2 file.zip!/miRDeep2 file/Know_Structure/LP42A2/oar-mir-539.pdf]

miRBase precursor : oar-mir-539
Total read count : 963
oar-miR-539-5p read count : 4
oar-miR-539-3p read count : 959
remaining reads : 0

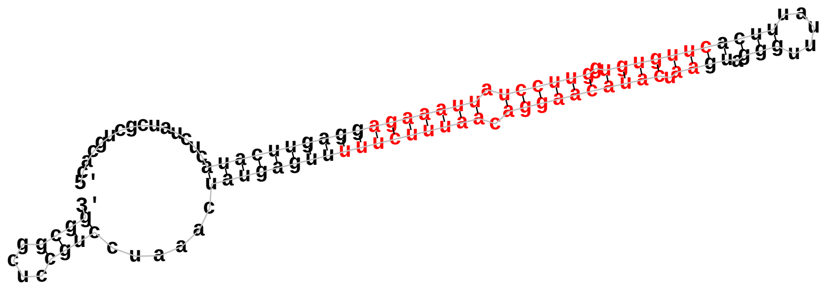

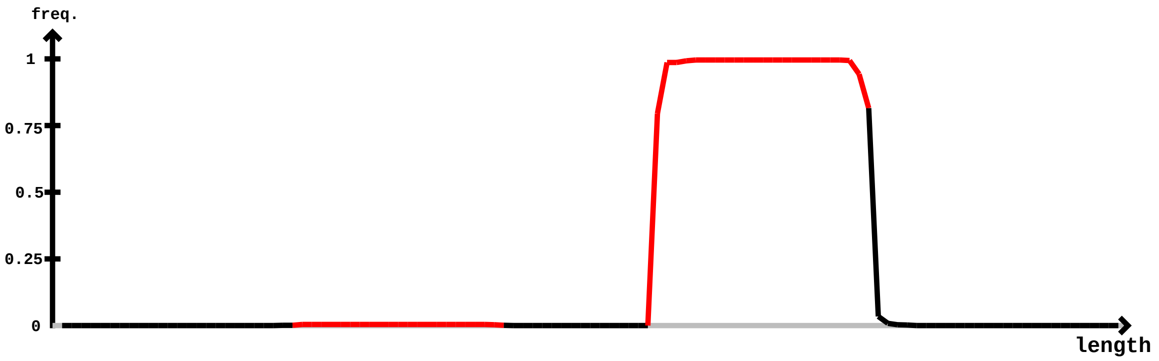


```
                                                             oar-miR-539-3p
                        oar-miR-539-5p
5'- cacgucgcuaucucauacuugaggagaaauuauccuugguguguucacuuuauuugggaugaaucauacaaggacaauuucuuuuugaguaucaaauccugccucggcgg   -3'   exp
    ..............(((((((((((((((((.((((((.(((((((((((.....))).)))).)))))))))).)))))))))))))))))......((((....))))   reads   mm   sample
    ......................ggagaaauuauccuuggugugu..................................................................   1       0    seq
    ........................agaaauuauccuuggugugguu.................................................................   2       0    seq
    ........................agaaauuauccuuggugugguuc................................................................   1       0    seq
    .............................................................aaucauacaaggacaauuuc.............................   2       0    seq
    .............................................................aaucauacaaggacaauuucu............................   48      0    seq
    .............................................................aaucauacaaggacaauuucuu...........................   116     0    seq
    .............................................................aaucauacaaggacaauuucuuu..........................   587     0    seq
    .............................................................aaucauacaaggacaauuucuuuu.........................   13      0    seq
    ..............................................................aucauacaaggacaauuucu............................   1       0    seq
    ..............................................................aucauacaaggacaauuucuu...........................   7       0    seq
    ..............................................................aucauacaaggacaauuucuuu..........................   165     0    seq
    ..............................................................aucauacaaggacaauuucuuuu.........................   11      0    seq
    ................................................................cauacaaggacaauuucuuuu.........................   1       0    seq
    ................................................................cauacaaggacaauuucuuuuu........................   5       0    seq
    .................................................................auacaaggacaauuucuuuuug.......................   1       0    seq
    .................................................................auacaaggacaauuucuuuuuga......................   2       0    seq
```
